# Bridging gaps in global surgery: Insights from an international hybrid conference

**DOI:** 10.1016/j.sopen.2025.02.002

**Published:** 2025-02-17

**Authors:** Fay Fathima Imtiaz Fareed, Leshanth Uthayanan, Robyn Anderson, Sai Kotecha, Adele Mazzoleni, Joshua Erhabor, Niraj S. Kumar, William Wong

**Affiliations:** a School of Medicine, University of Buckingham, Buckingham, United Kingdom; b InciSion UK, London, United Kingdom; c National Medical Research Association, London, United Kingdom; d St George's University of London, London, United Kingdom; e Nottingham City Hospital, Nottingham, United Kingdom; f Stoke Mandeville Hospital, Aylesbury, United Kingdom; g Barts and The London School of Medicine and Dentistry, London, United Kingdom; h St George's Hospital, London, United Kingdom; i Department of Cardiovascular Sciences, University of Leicester, Leicester, United Kingdom; j Watford General Hospital, Watford, United Kingdom

**Keywords:** Conference, Medical education, Global health, Surgery, Global surgery, Training

## Abstract

This study explores the motivations for attendance, methods of conference promotion, and key considerations in implementing a hybrid conference for global surgery education among medical students and trainee doctors. The InciSion UK Global Surgery Conference 2023, held in London, provided a unique platform combining in-person and virtual participation. Pre- and post-conference surveys collected qualitative and quantitative data from 640 and 794 respondents, respectively. Professional development (83.4 %) and learning about global surgery (70.8 %) were primary motivators for attendance. Confidence in global surgery significantly increased post-conference (mean score: 4.21 vs. 2.82, *p* < 0.001). Social media was the most effective promotional tool, introducing 38.4 % of attendees to the event. Participants highlighted the diversity and quality of talks as strengths, while technical issues were a challenge. Future conferences should enhance technical infrastructure and interactive engagement for virtual attendees. The hybrid model proves effective in broadening access to global surgery education and fostering collaboration across diverse geographical regions.

## Highlights

• This study included a robust sample size (794 responses), the largest to the author's knowledge.
• Using a pre- and post-study design allowed us to compare attendee confidence and knowledge of global surgery effectively.
• Qualitative and quantitative analysis was conducted to establish efficient promotion strategies for hybrid conferences and understand attendee perspectives.
• Attendees reported a significant improvement in understanding of global surgery as a result of the conference
• In future events, attendees most wanted to learn about “specific surgical and medical specialties” and “the state of surgery around the world”

## Introduction

Within the last decade, global surgery has been at the forefront of discussion in both low-middle-income countries (LMICs) and high-income countries (HICs). [1] There is a significant gap in access to quality surgical care between HICs and LMICs, with 90 % of LMICs lacking essential surgical care. [2] Previous literature has demonstrated the importance of multilevel collaborative partnerships inclusive of students, trainees, and institutions to develop an informed workforce capable of reducing surgical burden. [3]

Global surgery is overlooked within medical school curricula and surgical training. A previous cross-sectional survey found that only 10.6 % of undergraduate students had previous exposure to global surgery education, and 66 % of respondents believed that global surgery should be included within medical school curricula. [4]

Student-led conferences have demonstrated satisfaction and exposure to global surgery. [5] However, these events have had relatively low attendance and there is a lack of research into their views, motivations, and interests for attending. No study has explored the idea of a hybrid event to expand the reach of global surgery beyond the home country and encourage participants to present their research.

The InciSion UK Global Surgery Conference was a hybrid conference intended to fill this gap in global surgical education. Through analysis of the pre-and post-survey responses, this study aims to establish motivations for attendance, effective conference promotion methods, additional areas of interest for attendees and key considerations when implementing a hybrid approach.

## Methods

InciSion UK Global Surgery Conference was held at the Royal Colleges of Surgeons of England, London on September 9th, 2023 and featured a combination of virtual and in-person talks and one panel discussion. A variety of topics within global surgery were covered including: Advocacy, Research, Innovation, Collaboration, Ethics, Surgery in Conflict Regions, Training and Education. A selected number of virtual and in-person attendees were invited to present research. The conference was streamed virtually via MedAll and access was free for LMIC participants.

Event promotion targeted students and healthcare professionals from both LMICs and HICs. Instagram, Twitter, Facebook and LinkedIn pages of Incision UK and RCS England and relevant societies were primarily used. Incision UK's ambassador network was also utilised to promote to doctors and university students. On completion of the post-conference survey, attendees were awarded a certificate of participation with 5.25 Continuing Professional Development points from RCS England.

A pre-conference survey (Supplement 1) was sent via email to participants one day prior. This survey collected demographic information, pre-conference knowledge of global surgery, attendees interests in global surgery, and motivations for conference attendance. The post-conference survey (Supplement 2) was disseminated via MedAll and gathered information on engagement, confidence in global surgery, areas for improvement, and topics to include in future global surgery events. Qualitative data was collected from free text responses, and quantitative data was collected using Likert scale (5 = very helpful, 1 = not helpful at all).

Demographic data was presented in tabular format. Thematic analysis of qualitative survey data was independently conducted by two authors (FFIF and NSK) exploring conference strengths and potential areas of improvement. Quantitative analysis was conducted using a *t*-test for comparison of understanding of global surgery pre- and post-conference. Mean and 95 % confidence intervals (CI) were also generated to summarise respondent scores. All analysis was conducted using STATA 18.0 (StataCorp, College Station, TX).

Responses from attendees that were vague or left empty were excluded from data analysis.

## Results

There were 944 conference registrations. 880 individuals attended, with 67 attending in-person. 640 (response rate = 67.8 %) attendees completed the pre-conference survey, and 794 (response rate = 90.2 %) attendees completed the post-conference survey.

Of 640 pre-conference responses, 432 (67.5 %) were doctors and 196 (30.6 %) were medical students (Table 1). 87.3 % of doctors (*n* = 377) were in early stages of medical training (i.e. interns or residents). Responses came from 65 countries, with 43.9 % (*n* = 281) of responses from Europe, 30.6 % (*n* = 196) from Asia, and 21.6 % (*n* = 138) from Africa. The most responses were received from the: UK (*n* = 238), India (*n* = 85), Sudan (*n* = 50) and Nigeria (*n* = 48). 57.0 % (*n* = 365) of responses came from LMICs.

**Table 1:** – Occupation and training level of those who registered to the conference.

Type of Registration	N	%
School Student	2	0.3
Medical Student	196	30.6
Intern	50	7.8
Foundation Doctor/ Senior House Officer	327	51.1
Surgical Trainee	19	3.0
Resident	23	3.6
Clinical Fellow	5	0.8
Consultant Doctor	6	0.9
Doctor - Non-Specified Level	2	0.3
Other Healthcare Professional/ Student	9	1.4
Non-Healthcare Professional	1	0.2
Total	640	100

In the pre-conference survey, respondents mentioned they were most interested in “Education and Teaching” (74.7 %, *n* = 478), followed by “Research” (70.0 %, *n* = 448), “Professional Development” (83.4 %, *n* = 534) and “Learning about Global Surgery” (70.8 %; *n* = 453).

Social media was most effective in conference promotion, introducing 38.4 % (*n* = 246) of respondents, followed by the Incision UK or RCS England websites (28.8 %, *n* = 184), word of mouth (17.0 %, *n* = 109), and events platforms such as Eventbrite or MedAll (11.7 %, *n* = 75).

Post-conference survey respondents (*n* = 794) showed significant increase in confidence in global surgery (2.82 vs 4.21, *p* < 0.001). The content was rated 4.43 (95 % CI: 4.38–4.48), while talks and panel discussions were rated 4.33 (95 % CI: 4.28–4.38) and 4.26 (95 % CI: 4.21–4.32) for engagement. The format of the hybrid conference was rated 4.41 (95 % CI: 4.36–4.46).

Thematic analysis revealed the key positive themes from the responses were: quality and diversity of speakers, organisation of the event, hybrid format and the opportunity to present research (Table 2). Whereas, the key negative themes were: technical challenges for online attendees, time management, non-technical challenges and organisation of hybrid events, and delivery and content of the sessions (Table 3).

**Table 2:** Key positive themes from the conference.

Key Positive	Top Themes	Percentage	Number of responses
Quality and diversity of speakers	“Interesting panel on the impact of the Ukraine Conflict on medical education in ukraine” “Excellent range of speakers with vast experiences” “Global Trauma and Emergency Surgery talk was inspiring”	55.3 %	439
Organisation of the event	“Good inclusivity of online attendees throughout” “Well organised with good structure and content” “Great user interface being an online attendee”	15.9 %	126
Hybrid format	“Posters being available on Medall alongside in-person was great” “As it was hybrid, it was accessible globally and hence inclusive”	7.6 %	60
Opportunity to present research	“Great opportunity to present research and engage with a large audience from diverse backgrounds” “Ability to present and learn more about global surgery from other presenters”	3.9 %	31

**Table 3:** Key negative themes from the conference.

Key Negatives	Top Themes	Percentage	Number of responses
Technical challenges for online attendees	“Internet connectivity and freezing sometimes” “Flow was disrupted by online stream losing connection” “Low audio at times and perhaps better sound quality”	22.8 %	181
Time management	“Better time keeping as conference ran over scheduled time” “Equal time for questions for in-person and online”	12.1 %	96
Non-technical challenges and organisation of hybrid event	“Greater in-person attendance capacity” “Accommodate for lag between in-person and online attendees”	9.9 %	79
Delivery and content of sessions	“More workshops and interactive breakout rooms” “Talks from more speakers but lesser time per speaker”	6.3 %	50

Attendees were most interested in the following to be discussed in future global surgery events: “Surgical and Medical Specialities”, “Global Surgery and the State of Surgery around the World”, “Technology, Innovation and Sustainability” and “Getting Involved in Global Surgery”.

## Discussion

Our conference has improved understanding of effective methods for engaging a multidisciplinary audience and reported a significant increase in confidence in global surgery (2.82 vs 4.21, *p* < 0.001), in keeping with previous conferences. [5,6]

With 802 conference attendees from 65 countries, it is evident there is widespread interest in global surgery and that hybrid virtual event platforms provide a powerful means to reach a global audience. The high proportion of registrations from LMICs (57 %) highlights the value of developing events with accessibility in mind. Making global surgery events available virtually and free of charge to LMIC attendees provides an invaluable opportunity for stakeholders to collaborate to achieve global health equity. [7] Pre-conference data showed a majority of attendees were medical students or junior doctors (81.7 %), in line with other conferences [5,8].

Notably, we found success through incentives (certificates and CPD points). This especially attracts resident doctors for whom CPD serves an annual mandatory requirement. Considering these trainees already have initiative to pursue global surgery, the additional benefit of fulfilling nationally mandated training goals allows for dedicated allocation of trainee time towards skill development in global surgery, which was not previously possible in other similar conferences. In a previous conference only 17.4 % of attendees were junior doctors, and 67.3 % of attendees were medical students, as it was hosted by university societies where CPD points would not have held the same value. [6]

A key positive highlighted from post-conference feedback was the opportunity to present research that was absent in previous global surgery conferences, as it facilitated academic engagement in Global Surgery and rewarded it with prizes and certificates. Certificates and CPD points were awarded only after completion of post-conference feedback forms allowing us to achieve a high feedback response rate (90.2 %) from 880 attendees.

As the hybrid conference format becomes more widely adopted within the medical community [9,10], this provides not only new capabilities but also new challenges for both organisers and attendees. Platforms such as MedAll or Eventbrite may be used to promote global surgical events, 11.7 % of attendees learned about the conference through this method.

Key factors for a successful virtual conference gleaned from our post-conference survey included: providing interactive online spaces for virtual attendees to present research and engage with other attendees, allocating adequate opportunities for questions from virtual attendees, and ensuring clear communication and technical support for virtual attendees. Consideration should be given to technical aspects (such as internet connectivity and audiovisuals), time management (with equal opportunities for interaction for online and in-person attendees), ability to accommodate more in-person attendees and presence of more workshops and breakout rooms allowing for networking.

## Limitations

The post-conference survey did not distinguish between data from how attendees accessed the conference nor from different demographic groups. As such, valuable insights into differences in experiences may be missed. Additionally, the post-conference survey relied on subjective participant ratings of various aspects of the event, such as confidence levels, engagement, and helpfulness.

## Conclusion

This study demonstrates the effectiveness of a global surgery hybrid conference to engage attendees and develop their insight into global surgery. Future improvements should focus on technical infrastructure, engagement strategies, and sustainable access to resources. Acknowledging these factors will enable global surgery conferences to remain inclusive, effective, and impactful in fostering the next generation of global health professionals.

## CRediT authorship contribution statement

**Fay Fathima Imtiaz Fareed:** Writing – review & editing, Writing – original draft, Visualization, Methodology, Investigation, Formal analysis, Data curation, Conceptualization. **Leshanth Uthayanan:** Writing – review & editing, Writing – original draft. **Robyn Anderson:** Writing – review & editing, Writing – original draft. **Sai Kotecha:** Writing – review & editing, Writing – original draft, Formal analysis. **Adele Mazzoleni:** Writing – review & editing, Writing – original draft, Conceptualization. **Joshua Erhabor:** Writing – review & editing, Writing – original draft, Conceptualization. **Niraj S. Kumar:** Writing – review & editing, Writing – original draft, Visualization, Supervision, Project administration, Methodology, Formal analysis, Conceptualization. **William Wong:** Writing – review & editing, Writing – original draft, Methodology, Formal analysis, Conceptualization.

## Author contributions

FFIF, WW, JE, and AM designed the study. WW, FFIF, SK and NSK accessed the data and performed analysis. NSK, FFIF, LU, AM, SK and JE authored the draft manuscript. All authors reviewed analyses, revised the manuscript, and read and approved the final manuscript. FFIF had full access to the data and is the guarantor for the study.

## Patient and public involvement

Patients and/or the public were not involved in the design, or conduct, or reporting, or dissemination plans of this research.

## Ethics approval

This study was exempt from IRB review requirements, as per guidance from the NHS Research Ethics Committee, as it was a service evaluation of an InciSioN UK conference. Patients were informed of the usage of their anonymised survey data in this manuscript.

## Funding

NSK received funding support from the 10.13039/501100000274British Heart Foundation under a 4 year MRes/PhD DTP. No specific funding was received for conducting this study.

## Declaration of competing interest

The authors declare the following financial interests/personal relationships which may be considered as potential competing interests: Niraj S Kumar reports a relationship with British Heart Foundation that includes: funding grants. If there are other authors, they declare that they have no known competing financial interests or personal relationships that could have appeared to influence the work reported in this paper.

## Appendix A Supplementary data

Supplementary material Image 1
