# Supplementary material for: Bridging gaps in global surgery: Insights from an international hybrid conference
Source: Surg Open Sci. 2025 Feb 17;24:38–41. doi: 10.1016/j.sopen.2025.02.002 (PMC11880706; doi:10.1016/j.sopen.2025.02.002)
Supplement: Supplementary file 1 — Supplementary material [file mmc1.pdf]

Supplement 1: Pre-Conference Survey

1. What country are you from? (Free-text)
2. What stage of medical training are you in?(Drop-down list: School Student, Medical Student, Intern, Foundation doctor/SHO, Nurse, Surgical Trainee, Consultant, Other Healthcare Professionals)
3. What motivated you to attend this conference? (Drop-down list: Learning more about global surgery, Professional Development, Research Presentation, Networking Opportunities)
4. What aspects of global surgery are you most interested in? (Drop-down list: Research, Education and Teaching, Innovation, Collaboration)
5. How would you rate your knowledge of global surgery prior to the conference?(1-10)
6. What specific skills or knowledge related to global surgery do you hope to gain or enhance during this conference?(Free-text)
7. How did you hear about this conference? (Drop-down list: Instagram, Website, Facebook, LinkedIn, Twitter, Word of mouth, Eventbrite, MedAll, RCS England Bulletin, Other)
8. Have you ever been involved with the Royal Colleges of Surgeons of England? (e.g. surgical courses etc.) (Yes/No)

Supplement 2: Post-Conference Survey

1. How confident were you in the topic of Global Surgery before the conference? (1-5)
2. How confident were you in the topic of Global Surgery after the conference? (1-5)
3. How engaging did you find the talks? (1-5)
4. How engaging did you find the panel? (1-5)
5. How helpful was the content overall? (1-5)

6. How interesting did you find the format? (1-5)
7. What went well? (Free-text)
8. What could have been better? (Free-text)
9. What further topics within Global Surgery would you like to learn about? (Free-text)
10. Would you like to be contacted by RCS England about future global events and opportunities? (Yes/No)
11. Would you like to be contacted by Incision about future global events and opportunities (e.g. research, education, advocacy projects)? (Yes/No)
